# Additive Influence of Top Metal Contact and Alumina Deposition on the Threshold Voltage of Suspended Carbon Nanotube Field-Effect Transistors

**DOI:** 10.1021/acsomega.3c03602

**Published:** 2023-07-19

**Authors:** Kishan Thodkar, Miroslav Haluska, Christofer Hierold

**Affiliations:** Micro- & Nanosystems, Department of Mechanical and Process Engineering, Tannenstrasse 3, ETH Zurich, 8092 Zurich, Switzerland

## Abstract

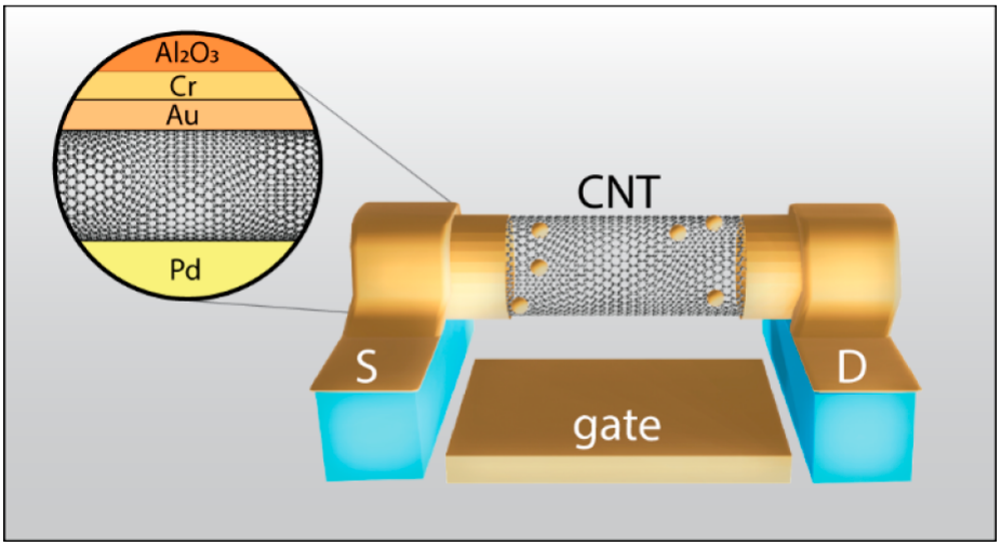

One-dimensional nanostructures such as carbon nanotubes offer excellent properties useful for applications in gas sensors, piezoresistive devices, and radio frequency resonators. Considering their nanoscale form factor, carbon nanotubes (CNTs) are highly sensitive to surface adsorbents. This study presents the fabrication flow of CNT devices with extended passivated areas around electrical contacts between the CNT and source and drain electrodes. These types of structures could help in understanding the intrinsic CNT response by eliminating the analyte impact on the Schottky barrier regions of the CNT field-effect transistors (CNTFETs). The influence of multiple processing conditions on the electronic properties of CNTFETs with a suspended individual CNT used as the CNTFET channel is presented. Our findings show a threshold voltage shift in CNT *I*_SD_–*V*_g_ characteristics following the metal deposition and alumina atomic layer deposition.

## Introduction

Carbon nanotubes offer a broad range of electronic, mechanical, and optical properties, making them interesting for various applications.^1−3^ Several applications of carbon nanotubes (CNTs) as transducers to measure and characterize strain, gas analytes, and electromechanical resonance have been demonstrated.^4−8^ Gas sensors fabricated from CNT-based transistors offer unique advantages such as ultralow power consumption^7^ and low detection limit of specific gaseous analytes. At a system level, these performance metrics are particularly advantageous for nanoscale gas sensors, especially within the context of a highly distributed network of Internet-of-things (IoT)-based sensors and electronic devices.^9−11^ Dry transfer of individual CNTs has been successfully demonstrated to provide a stable platform to manufacture ultraclean, high-performance CNT devices.^12,13^ The devices realized with this approach offer a photoresist-free approach for the integration of CNTs into devices in bottom-metal contact architecture.^12,13^ In such a configuration, the CNT is anchored on top of the metal electrode and exposed to ambient conditions. In such a scenario, the CNT region on the metal electrodes remains exposed to ambient gas molecules. This can directly influence the Schottky barrier at the metal electrode–CNT interface and, thereby, the CNT field-effect (CNTFET) characteristics.^14,15^

In this work, we demonstrate the photolithography-free passivation of CNTFETs. As-grown suspended CNTs were first localized using Raman spectroscopy on growth substrates (oxidized SOI chips with interbeam trenches of ∼10–12 μm) and transferred to receiving substrates with preprepared FET device architecture. The dry transfer was followed by exposure of the CNTFETs to six processing conditions. After every processing step, electrical CNTFET characterization was performed. Chips were stored (during the ten-day stability test) under cleanroom conditions (45 ± 5% relative humidity, 20–23 °C, ambient pressure). Scanning electron microscopy (SEM) characterization was performed at the end of the study. CNTFET threshold voltage and current on/off ratio were characterized during the multiple processings.

## Results

**Figure 1:**
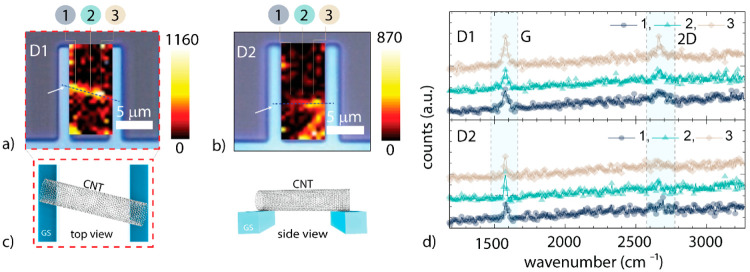
Localization of CNTs on the growth substrate performed using Raman spectroscopy. (a,b) G-band image of CNTs D1 (a) and D2 (b) overlaid on an optical image of the growth substrate. The color bar represents the G-peak intensity count. (c) The top and side view illustration of the suspended CNT on a growth substrate (GS). (d) Raman spectra from the three regions on the CNT. Note: The conditions used for the characterization are objective: 50× (NA = 0.75). Wavelength: 514 nm. Laser power: 5 mW. Exposure time: 0.1 s. Five accumulations.

In Figure 1, we show the localization of suspended CNTs on the growth substrate between Si beams by using Raman spectroscopy. In Figure 1(a–d), the G-band intensity images are overlaid on optical images of the growth substrate (top view). An illustration of a suspended CNT on the growth substrate (GS) is shown in Figure 1a. The G-band intensity depicts the suspended CNTs across the trench structure, indicated by using dotted lines. The representative Raman spectra at the three regions are shown in Figure 1c.^16^

**Figure 2:**
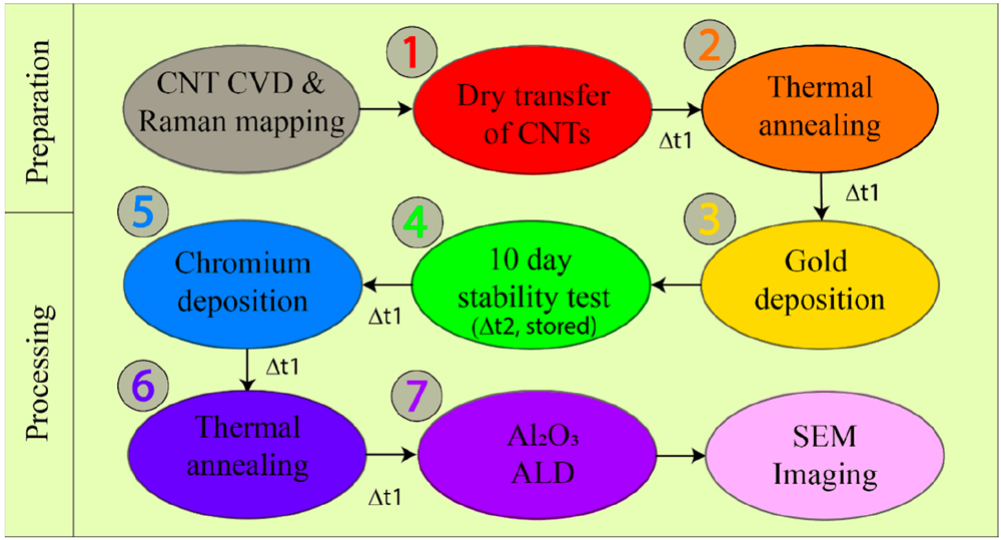
Overview of the process flow followed under class 100 cleanroom conditions during this work. Note: the dry transfer of CNTs is performed on device substrates with Pd bottom metallization: Δt1 denotes < 30 min, and Δt2 denotes 10 days.

An argon ion etching was performed on the device substrates to remove potential contaminants from the source and drain electrode surfaces. This was followed with the dry transfer of CNTs D1 and D2 onto device substrates between Pd metal electrodes; additional details related to the influence of argon ion etching on the CNTFET characteristics can be found in our recent work by S. Jung et al.^13^ In Figure 2, an overview of the seven processing steps that were performed on the same CNTFETs is presented. First, the CNTs are transferred from growth substrates onto device substrates following the ultraclean dry transfer process. Second, to improve the adhesion at the metal–CNT contact interface, 60 min thermal annealing at 300 °C is performed under nitrogen flow at 10 mbar. By following the two steps, suspended CNTFETs with palladium metal electrodes as bottom source (S) and drain (D) contacts were realized. The Pd gate electrode was located 2 μm below the surface of the S & D electrodes. Note that palladium (ϕ_Pd_ ∼ 5.2 eV, ϕ is the work function) metal electrodes are preferred due to their low Schottky barrier (Ψ) contact properties to CNT at CNFETs.^17^

In bottom-contact CNTFETs, the Pd–CNT contact region is exposed to environmental conditions. Complete passivation of the Pd–CNT contact interface is of high interest for sensor applications. Here, we report a top metallization study utilizing gold, expected to form a low Schottky barrier height difference Ψ_BP_ with the CNT (ϕ_CNT_ ∼ 5.05 eV). This was realized with the deposition of five-nanometer gold (ϕ_Au_ ∼ 5.1 eV). Gold was preferred as the first top metal layer due to the weak Au–carbon interaction, with minimal risk of forming a continuous film covering the entire CNT, as reported by Zhang et al.^18^ In contrast to gold, metals such as chromium and titanium are commonly used as adhesion-enhancing metals with a strong metal–CNT interaction.^18^ However, chromium layers have been studied to enhance the ALD deposition of Al_2_O_3_.^19^

First, a five-nanometer gold layer was deposited using thermal e-beam evaporation (see step 3, Figure 2). Note: The metal deposition process was performed without a photolithographic mask. After a stability test at ambient conditions (duration: 10 days), a five-nanometer chromium layer was deposited (see step 5, Figure 2), without a mask.^18,20^ During the sixth processing step, the second round of thermal annealing was performed. The last step consists of the atomic layer deposition of Al_2_O_3_. This is performed to passivate the metal contact regions with a dielectric passivation layer. The suspended CNT region remains uncovered by the passivation layer.

**Figure 3:**
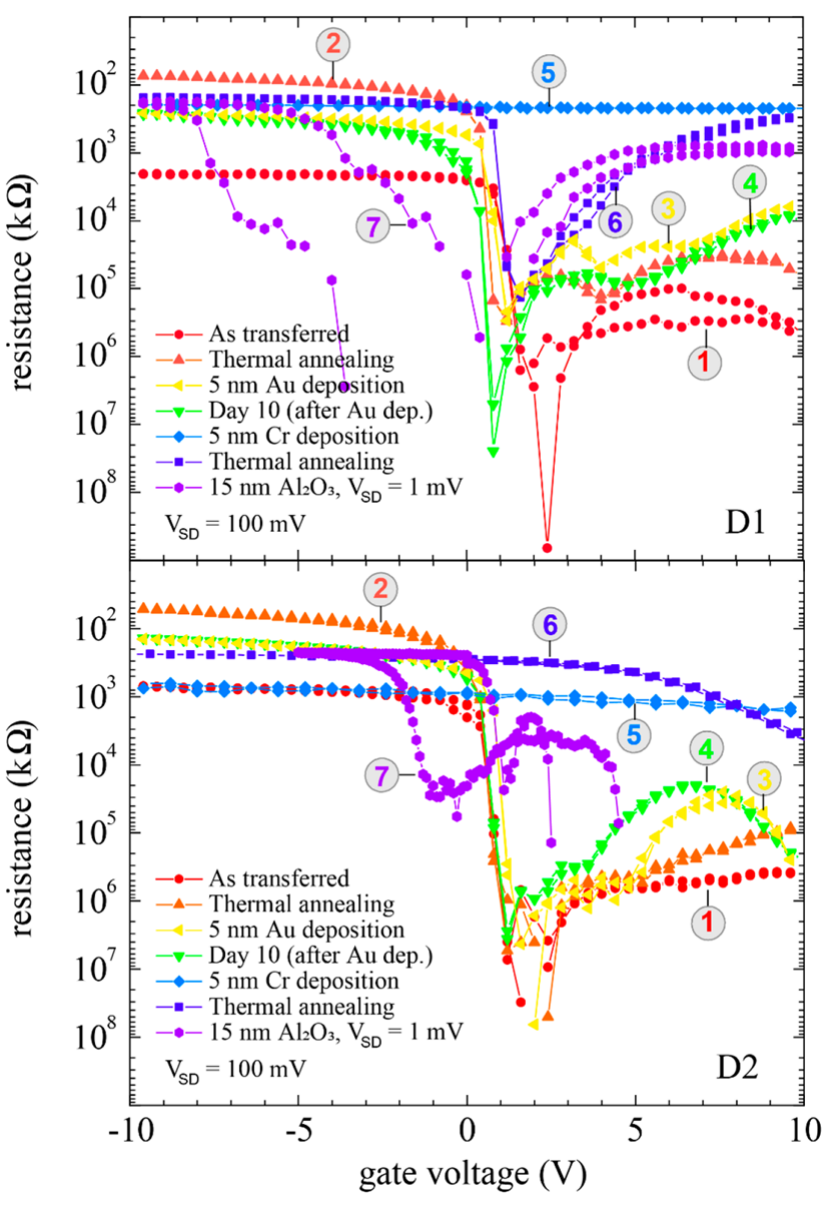
Field-effect characterization of two CNTFETs after individual processing steps. Resistance (kΩ) vs gate voltage (*V*) characteristics of as-transferred (red circle, No. 1), thermal annealed (orange triangle, No. 2), 5 nm gold deposited (light orange rotated triangle, No. 3) after 10 days (light green inverted triangle, No. 4), 5 nm Cr deposited (dark yellow rotated square, No. 5), post 2nd thermal annealing (light yellow square, No. 6), and after 15 nm Al_2_O_3_ ALD process (green hexagon, No. 7). Note: The characteristics were measured under ambient cleanroom conditions of temperature 20–23 °C, ambient pressure (1 bar), and 45 ± 5% relative humidity.

After every processing step, we captured the FET characteristics to study the impact of the processing step on the device performance. Note that all the processing steps and sample storage conditions were in class 100 cleanroom conditions. The time window between the processing steps was under 30 min. All seven FET characteristics of the CNTFETs D1 and D2 after each processing step are presented in Figure 3. The first FET characteristic was measured after performing the dry transfer of CNTs onto the device substrates (Figure 3, red circle). After thermal annealing (300 °C, 10 mbar nitrogen flow, 60 min), we observed an improvement in field-effect characteristics (Figure 3, pale red triangle). This is attributed to the decrease of electrical contact resistance at the metal electrode–CNT interface. The field-effect characterization performed after the thin gold film deposition seems to be influenced by the thermal evaporation step (Figure 3, curve Nr. 3). The time between thermal evaporation and electrical characterization was under 15 min.

After the Au thermal evaporation step, we performed a time stability test and remeasured the CNTFET characteristics after 10 days. The CNTFETs were stored in an ESD safe storage box under cleanroom conditions (45 ± 5% RH, 20–23 °C, ambient pressure). The characterization shows that the FET characteristics remain intact with no sign of degradation (Figure 3, olive green, inverted triangle). These observations were consistent in both the CNTFETs. After the five-nanometer thick chromium layer was deposited, metallic characteristics were observed in the devices (Figure 3, dark yellow rotated squares). This is indicative of a continuous Cr film connecting the S & D electrodes. However, the FET characteristics were recovered after a second thermal annealing process, in the case of CNTFET D1 (Figure 3, yellow square) but not for CNTFET D2. The recovery of the characteristics can be due to the formation of a noncontinuous chromium layer on the CNT after the thermal annealing process. During the ALD process of Al_2_O_3_ deposition (at *T* = 300 °C) one can expect that Al_2_O_3_ is deposited on the Cr layer and not on the CNT surface. There was an increase in hysteretic behavior (Figure 3, green hexagon) after the ALD process was observed. To minimize device failure after ALD, the characterization at a potential bias (*V*_SD_) ∼ 1 mV and reduced gate voltage sweep window was considered. A comparison of the CNTFET characteristics after each processing step is presented in Supporting Information, Figures S1 and S2.

**Figure 4:**
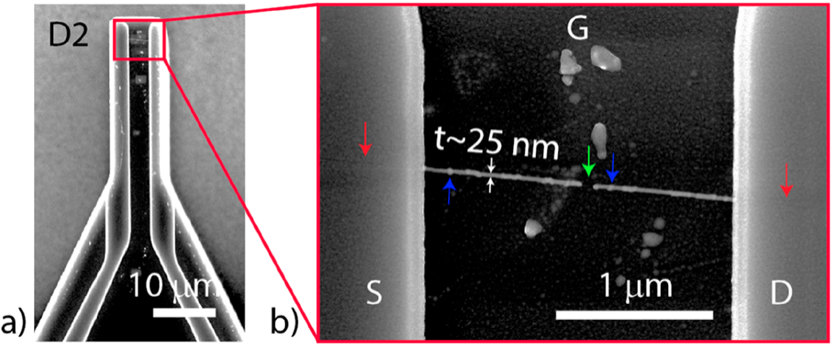
Scanning electron microscope image of the CNTFET D2 taken postprocessing. The CNT is highlighted by using arrows. (a) Overview of the two-terminal CNTFET with the CNT highlighted within the red box. (b) SEM of the suspended CNT after postprocessing depicting high surface coverage of the CNT surface with the unpassivated region highlighted using a green arrow. The estimated thickness (*t*) of the passivation is ∼25 nm. Note: The presence of beads on the CNT is highlighted using blue arrows.

We imaged the CNTFETs using scanning electron microscopy (SEM) after the final FET measurements. In Figure 4, the SEM images of CNTFET D2 are presented. Complete passivation of the CNT region on the metal electrode regions is highlighted by using red arrows. From Figure 4b, we can estimate the deposition thickness of ∼25 nm. Several beads along the CNT are observable, as highlighted by using the blue arrows. The bead thickness is ∼50 nm. The SEM characterization of D1 is presented in Supporting Information Figure S3.

## Discussion

**Figure 5:**
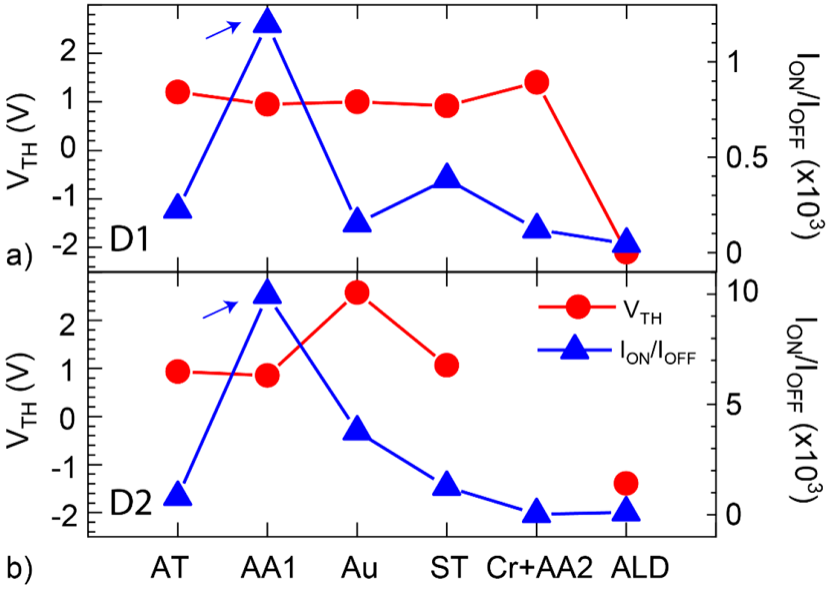
Influence of processing steps on the threshold voltage (*V*_TH_, red circle) and current on/off ratio (*I*_on/off_, blue triangle) of D1 (a) and D2 (b). The blue arrow highlights the increase in the *I*_on_/*I*_off_ ratio after the thermal annealing process. Note: As transferred (AT), thermal annealing 1 (AA1), gold deposition (Au), stability test (ST), chromium deposition and thermal annealing 2 combined (Cr + AA2), and atomic layer deposition (ALD). The data point for D2 after the Cr + AA2 process is excluded due to metallic characteristics.

In Figure 5, we present the threshold voltage (*V*_TH_, red circle) and the current on/off ratio (*I*_on/off_, blue triangle) extracted from the FET characteristics presented in Figure 3. A significant increase in the *I*_on/off_ ratio after the thermal annealing process is observable in both the CNTFETs. We primarily attributed this to the reduction in contact resistance at the metal–CNT contact interface during the annealing process. We observe a decrease in the *I*_on/off_ ratio after the thermal deposition of a five-nanometer gold film. After 10 days, we repeated the field-effect measurements and found no further decrease compared to measurements following the gold deposition. Such measurements after a prolonged time are helpful in the detection of possible device degradation.

However, an incremental decrease in the *I*_on_/*I*_off_ ratio was observed after each of the following processing steps. Such a decrease in *I*_on_ can occur due to enhanced charge scattering caused by gold nanoparticles or beads formed on the CNT surface. The CNTFET characteristics remain stable on repeating the electrical characterization 10 days after the gold deposition process, presented in Supporting Information Figure S1c,d. The threshold voltage (*V*_TH_) was extracted from the CNTFET characteristics. During the processing conditions, namely, as-transferred (AT), thermal annealing 1 (AA1), gold deposition (Au), and stability tests at the ambient conditions (ST), the *V*_TH_ is at positive gate voltages (*V*_TH_ ∼ 1 V). The *V*_TH_ after gold deposition is comparable to *V*_TH_ after the first thermal annealing step. This can be understood by the Schottky barrier at the metal–CNT interface. The work function (ϕ_m_) of single-walled CNTs is ∼5.05 eV, palladium ∼5.2 eV, and gold ∼5.1 eV.^21^ The Schottky barrier height (Ψ_Bp_) for holes can be estimated by considering Ψ_Bp_ = *E*_gCNT_ + χ_CNT_ – ϕ_metal_,^22^ where *E*_gCNT_ is the bandgap, χ_CNT_ the electron affinity, and ϕ_metal_ the work function of the metal electrode. Considering the χ_CNT_ of ∼4.7 eV,^23^ ϕ_Pd_ ∼ 5.2 eV,^24^ and an *E*_gCNT_ bandgap of ∼0.4 eV,^25^ Ψ_Bp_ can be estimated to be ∼−0.1 eV.

Minimal Ψ_Bp_ with Pd contacts supporting hole conduction has been well studied and commonly used as a metal electrode during CNTFET fabrication. Chen et al. have reported on the CNTFET on-current as a function of CNT diameter and Schottky barrier for palladium, titanium, and aluminum metal contacts using an extended Schottky barrier model.^17^ In addition, note the gradual increase in the off current and a decrease in the on current in Figure 3a,b, which is primarily attributed to the change in work function at the metal–CNT interface.^26^ Note that the CNT is sandwiched between palladium (bottom layer) and gold (top layer) at the contact region, which can lead to a change in the work function at the metal–CNT interface.

A metallic behavior can be observed in CNTFET characteristics after 5 nm Cr layer deposition (see curve 5, Figure 4a,b). Liu et al. reported on the observation of thin Cr film formation on suspended CNTs, starting with a deposition thickness of ∼0.4 nm using TEM.^27^ However, after thermal annealing, semiconducting CNTFET characteristics are present in D1, but D2 exhibits a weak field effect at a gate voltage >5 V (see Figure S2d, yellow square). From the SEM images, we can notice the suspended CNT region without metal or Al_2_O_3_ (Figure 4b, green arrow). This is indicative of the fact that the Cr film formed on the CNT is influenced by the thermal annealing step.

Post Al_2_O_3_ ALD, hysteretic FET characteristics are observable in D1 and D2. The *V*_TH_ modulation after thermal annealing (AA2, see Figure 5) and the ALD process suggests the influence of charge traps on the CNTFET characteristics. In D1 and D2, the *V*_TH_ after the ALD process is at a negative gate voltage (*V*_TH_ ∼ −2 V, extracted from the forward FET trace). Moreover, the device characteristics show significant hysteretic behavior after the ALD process (see: curve no. 7, Figure 5). The hysteretic effect can be attributed to the enhanced trapping of charges at the Al_2_O_3_ dielectric layer. The hydrophilic nature of Al_2_O_3_ can improve the adsorption of water molecules when the CNT is exposed to ambient conditions.

Such observations have also been made in CNTFETs wherein ALD was performed directly after the fabrication of the bottom-contacted CNTFET device, presented in Supporting Information Figure S4 curve no. 3.^15,28^ Note that the work function of the CNT can be influenced under such processing conditions. A decreasing work function of (MW) CNTs from ϕ_CNT_ ∼ 4.8 eV to ϕ_CNT_ ∼ 3.3 eV on exposure to hydrogen-containing plasma has been reported by Bulyarskiy et al.^29^ Such a change in the work function can influence the overall Ψ_Bp_ and CNTFET characteristics.

To support these discussions, SEM characterization after extensive processing conditions is beneficial. In Figure 4b, we can see the suspended part of CNTFET D2 passivated with a thickness of ∼25 nm (estimated from the SEM image). During the ALD process, Al_2_O_3_ was deposited with a thickness target of *t*_ALD_ ∼ 15 nm. Considering wrap-around passivation of the CNT, the net thickness *t*_net_ ≈ 2*t*_ALD_ + CNT_diameter_ ≈ 32 nm (where CNT_diameter_ ∼ 2 nm). We can also observe several beads with thickness *t*_bead_ > *t* (to be ∼50 nm), which can contribute to charge scattering, as highlighted during the earlier discussions and reported by Zhang et al.^18^

Moreover, the possibility of gold and chromium beads is highly likely after the thermal evaporation of gold and chromium, followed by thermal annealing.^30^ Our findings provide detailed insight into the influence of different processing conditions that follow the fabrication of CNTFETs. We study the influence of two photolithography maskless techniques to deposit thin metal films and Al_2_O_3_ ALD directly on CNTFETs. First, thin-film deposition of gold and chromium on bare CNTFETs can be used to form top metal contact with CNTFETs with minimal hysteresis. Second, the influence of the ALD deposition of Al_2_O_3_ on CNTFET was characterized.

## Conclusion

In conclusion, the additive effects of processing conditions on the electrical characteristics of the CNTFET were studied. A decrease in the *I*_on_/*I*_off_ ratio was observed on the deposition of the metal layer and Al_2_O_3_ ALD on CNTFETs. Metallic characteristics observed after chromium deposition were reversed back to semiconducting characteristics following a thermal annealing process. By depositing thin metal and dielectric films, the metal–CNT contact regions are covered. This approach helps in minimizing the influence of analyte interaction at the metal–CNT interface.

## Experimental Section

### Fabrication, Transfer, and ALD

Prepatterned receiving substrates with FET architecture were fabricated using MEMS processes.^13^ 1 nm chromium (Cr) and 40 nm palladium (Pd) were deposited using electron beam deposition to form the source (S), drain (D), and gate (G) electrodes for the CNTFETs. Next, the argon ion sputtering (120 s, 13 W) step was performed to prepare the electrodes for the transfer. CNTs were grown using the chemical vapor deposition (CVD) process at a growth temperature of ∼850 °C, with CH_4_ 300 sccm and H_2_ 200 sccm at 500 mbar, using ferritin as a catalyst precursor.^31^ After the CVD process, the CNTs were transferred from the growth substrates onto the receiving substrates by using the dry transfer method. The growth substrate consists of forklike structures at the edge of the substrate. After the CNT synthesis, the CNTs are suspended across the fork structures, as observable in Figure 1. The device substrate (DS) consists of source (S) and drain (D) electrodes with a channel length of ∼2.8 μm and gate (G) distance of ∼1 μm. The device substrate (DS) is bonded to a ceramic package using silver paste, and then using aluminum wire bonding the S/D/G electrodes are connected to the package. Package with DS is interfaced with a readout PCB and measurement instrumentation (Keithley source meter). The growth substrate (GS) is placed on a micromanipulator with XYZ positioning. The suspended CNTs on fork structures on the GS is aligned with DS, and mechnical transfer of CNTs from GS to DS is performed, as described by Mouth et al.^32^ On successful transfer of a CNT, the CNTFET characteristics can be acquired using the measurement instrumentation.^13,32^ After the transfer process, a thermal annealing process was performed at 300 °C for up to 60 min at 10 mbar of nitrogen flow. The electrical measurements were performed using two Keithley source meters. The gate voltage sweep was performed within a ±10 V range with 400 mV per step in a continuous procedure (*V*_SD_ = 100 mV). Five-nanometer gold and chromium deposition (5 nm) was performed using electron beam deposition (deposition rate ∼ 0.05 Å/s). ALD (15 nm) of Al_2_O_3_ was performed using the Picosun ALD deposition tool at *T* = 300 °C.

### Raman Spectroscopy Characterization

The CNTs were characterized using an inVia Renishaw confocal microscope using a 514 nm excitation wavelength. The spectral acquisition conditions were: objective 50× (NA = 0.75); wavelength 514 nm; laser power 5 mW; exposure time 0.1 s, and 5 accumulations using a grating with 600 grooves per millimeter.

## Supporting Information Available

CNTFET characteristics after each processing step are presented in figures S1 and S2. The SEM characterization of CNTFET D1 is provided in figure S3. Comparison of the CNTFET characteristics before and after 15 nm Al_2_O_3_ ALD deposition is presented in figure S4 (PDF)
