# Supplementary material for: Additive Influence of Top Metal Contact and Alumina Deposition on the Threshold Voltage of Suspended Carbon Nanotube Field-Effect Transistors
Source: ACS Omega. 2023 Jul 19;8(30):27697–702. doi: 10.1021/acsomega.3c03602 (PMC10398841; doi:10.1021/acsomega.3c03602)
Supplement: Supplementary file 1 — ao3c03602_si_001.pdf [file ao3c03602_si_001.pdf]

Supporting Information

Additive Influence of Top Metal Contact and Alumina Deposition on Threshold Voltage of Suspended Carbon Nanotube Field-Effect Transistors

Kishan Thodkar, Miroslav Haluska, and Christofer Hierold*

Micro- & Nanosystems, Department of Mechanical and Process Engineering,

Tannenstrasse 3, ETH Zurich, 8092 Zurich, Switzerland

*e-mail: kishant@ethz.ch

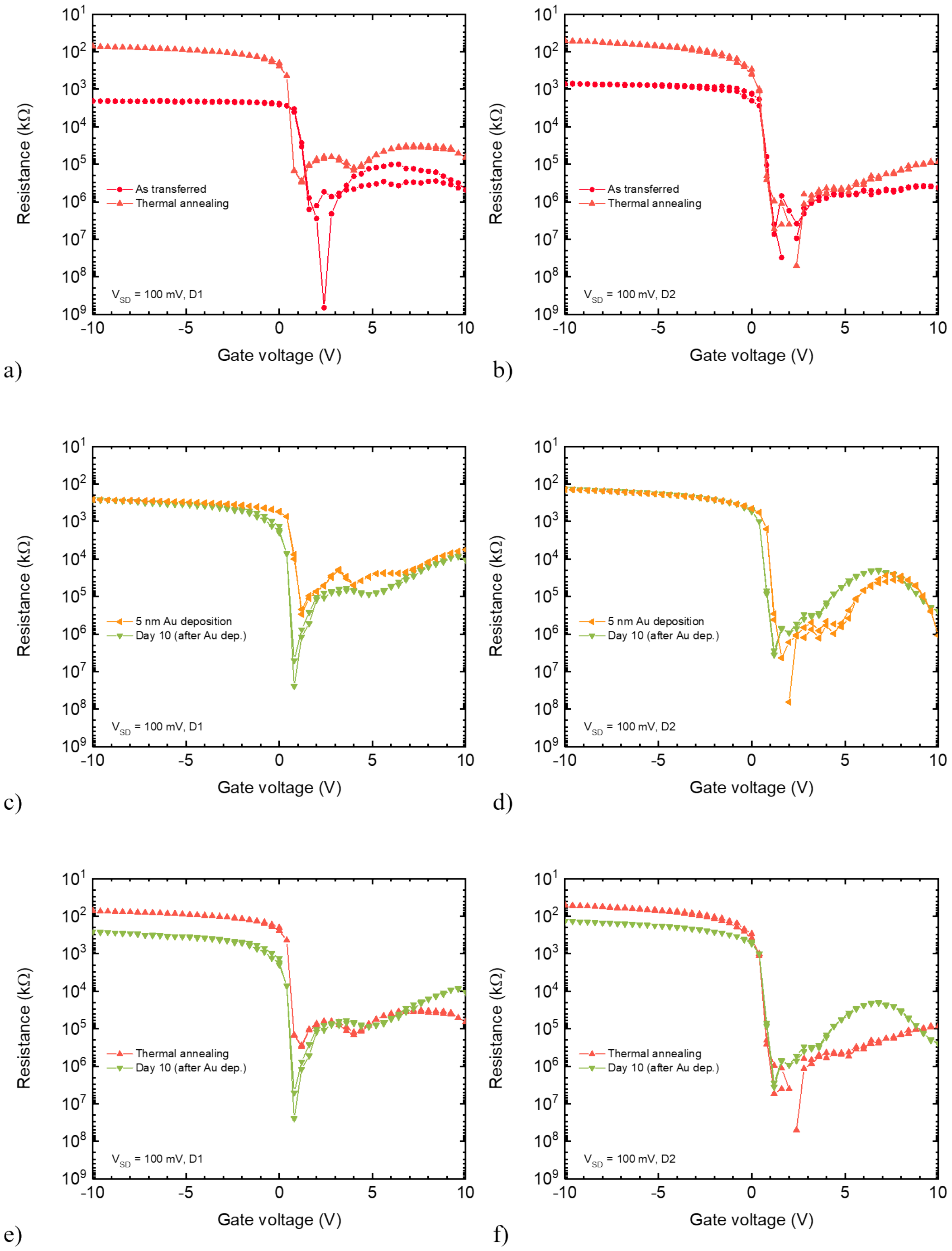

Figure S1. *Comparison of CNTFET characteristics of D1 and D2. Resistance (kΩ) vs. gate voltage (V) characteristics of* ***a-b)*** *as-transferred (red circle) and thermal annealing (orange triangle).* ***c-d)*** *After five-nanometre gold deposition (light orange left-rotated triangle) and ten days after gold deposition (light green inverted triangle).* ***e-f)*** *After thermal annealing (orange triangle) and ten days after gold deposition (light green inverted triangle).*

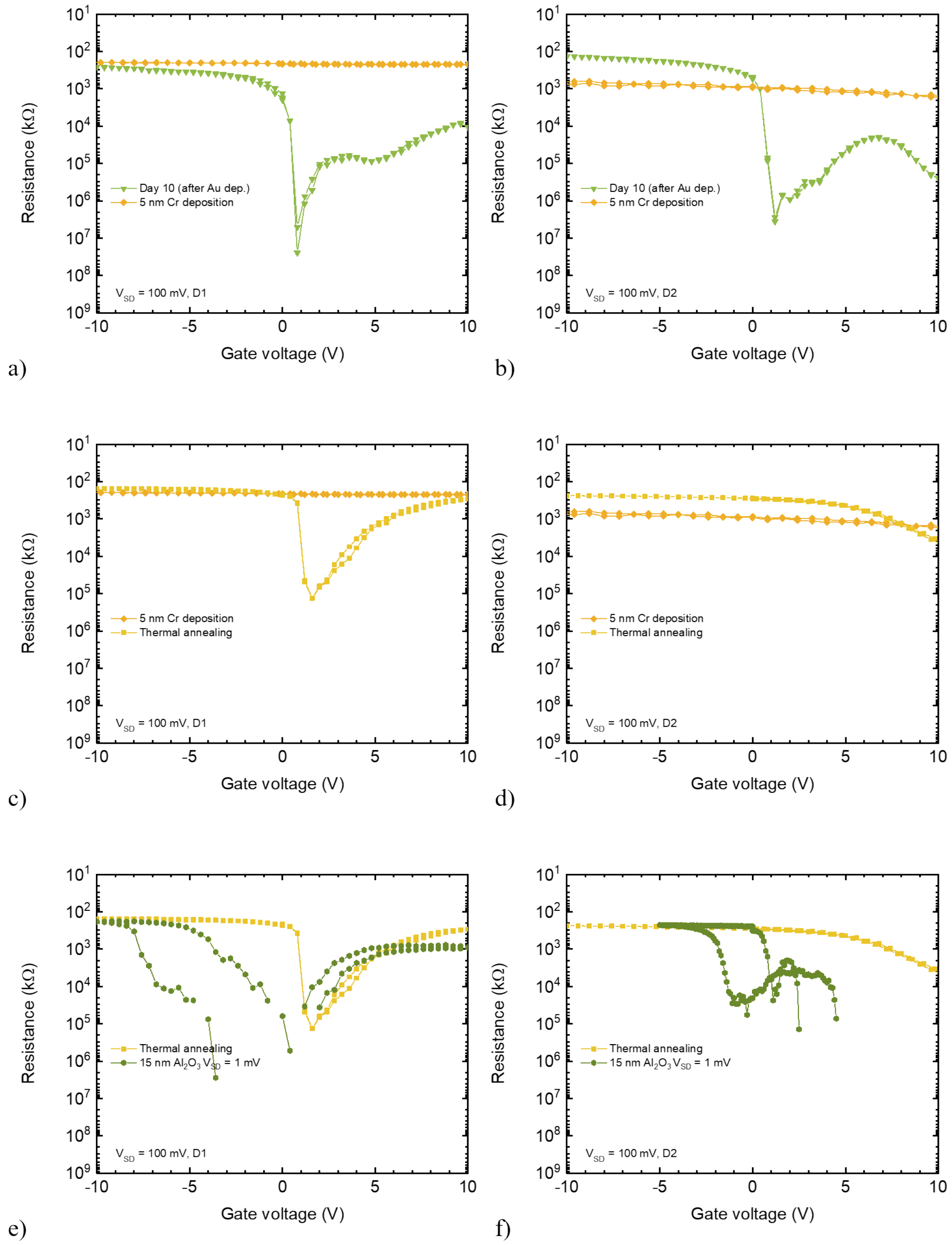

Figure S2. *Comparison of CNTFET characteristics of D1 and D2. Resistance (kΩ) vs. gate voltage (V) characteristics* ***a-b)*** *ten days after gold deposition (light green inverted triangle)transfer (re, circle) and five-nanometre chromium deposition (dark yellow inverted square).* ***c-d)*** *After five-nanometre chromium deposition (dark yellow inverted square) and thermal annealing (light yellow square).* ***e-f)*** *Thermal annealing (light yellow square) and 15 nm Al_2O_3 ALD (green hexagon).*

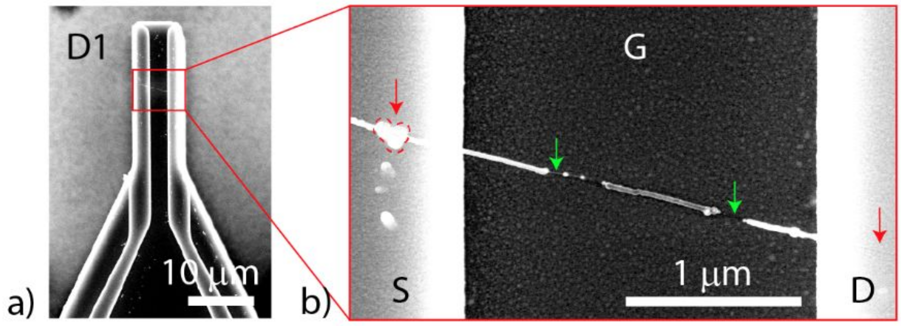

Figure S3. *Scanning electron microscope image of the CNTFET D1 taken post-processing. The CNT is as highlighted using the arrows. a) Overview of the two-terminal CNTFET with the CNT highlighted within the red box. b) SEM of the suspended CNT post-processing depicting surface coverage of CNT surface with the two un-passivated regions highlighted using the green arrows. The areas of CNT passivated at the metal electrode region is highlighted using red arrows.*

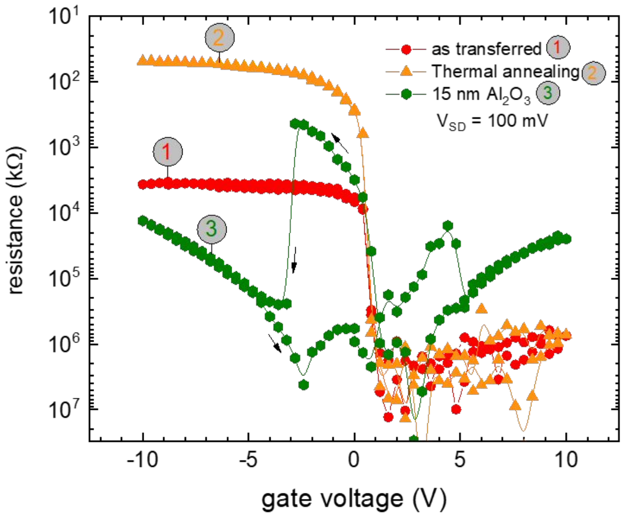

Figure S4. *Comparison of CNTFET characteristics of KTDS3-D4 sample with 15 nm Al_2O_3 ALD performed directly after the thermal annealing process. Resistance (k**Ω**) vs. gate voltage (V) characteristics of as transferred (red circle, curve Nr. 1), after thermal annealing (orange triangle, curve Nr. 2) and 15 nm Al_2O_3 ALD deposition (green hexagon, curve Nr. 3).* ***Note:*** *The black arrows indicate the upward sweep direction and the presence of hysteresis after Al_2O_3 ALD deposition.*
